# Whole-genome and dispersed duplication, including transposed duplication, jointly advance the evolution of *TLP* genes in seven representative Poaceae lineages

**DOI:** 10.1186/s12864-023-09389-z

**Published:** 2023-05-30

**Authors:** Huilong Chen, Yingchao Zhang, Shuyan Feng

**Affiliations:** 1 College of Life Sciences, North China University of Science and Technology, Tangshan, 063210 Hebei China; 2 College of Grassland Science and Technology, China Agricultural University, Beijing, 100193 China

**Keywords:** Transposed duplication, Codon bias, Selection pressure, Synteny network, Tubby-like protein, Evolution

## Abstract

**Background:**

In the evolutionary study of gene families, exploring the duplication mechanisms of gene families helps researchers understand their evolutionary history. The tubby-like protein (TLP) family is essential for growth and development in plants and animals. Much research has been done on its function; however, limited information is available with regard to the evolution of the *TLP* gene family. Herein, we systematically investigated the evolution of *TLP* genes in seven representative Poaceae lineages.

**Results:**

Our research showed that the evolution of *TLP* genes was influenced not only by whole-genome duplication (WGD) and dispersed duplication (DSD) but also by transposed duplication (TRD), which has been neglected in previous research. For *TLP* family size, we found an evolutionary pattern of progressive shrinking in the grass family. Furthermore, the evolution of the *TLP* gene family was at least affected by evolutionary driving forces such as duplication, purifying selection, and base mutations.

**Conclusions:**

This study presents the first comprehensive evolutionary analysis of the *TLP* gene family in grasses. We demonstrated that the *TLP* gene family is also influenced by a transposed duplication mechanism. Several new insights into the evolution of the *TLP* gene family are presented. This work provides a good reference for studying gene evolution and the origin of duplication.

**Supplementary Information:**

The online version contains supplementary material available at 10.1186/s12864-023-09389-z.

## Introduction

Genes can be duplicated through a variety of mechanisms, including whole-genome duplication (WGD), tandem duplication (TD), proximal duplication (PD), dispersed duplication (DSD), and transposed duplication (TRD). Exploring the different duplication mechanisms of gene families helps us understand the origin and evolution of the genes and provides unique insights. By identifying the types of duplication origins of cold resistance genes in plants, Song et al. found that cold resistance genes can originate from singletons, DSD, PD, TD, and/or WGD and that WGD and DSD were the major contributors to gene duplication, thus proposing the hypothesis that cold resistance genes were preferentially retained after polyploidization events [1]. Liu et al. found that both WGD and TD played an important role in the expansion of intronless genes in intron-poor subfamilies by identifying the type of origin of duplication of intron-poor and intronless family genes in plants [2]. Nezamivand‑Chegin et al. proposed that WGD or DSD types were the most frequent contributors to SPX expansion by identifying the duplication types of SPX family genes in plants [3]. Therefore, it is useful to perform an analysis of the type of duplication origin of genes in evolutionary studies of gene families.

These duplication types are recognized by the duplicate_gene_classifier program in the MCScanX package and are divided into five types: singleton, WGD, TD, PD, and DSD [4, 5]. MCScanX enforces the determination that the origin of duplication of a gene can belong to only one of the five duplication types and does not allow a gene to originate through different duplication mechanisms. However, the true meaning of a singleton, as mentioned above, is that it does not undergo duplication; i.e., it should not be called a type of duplication. There are five types of gene duplication mechanisms: WGD, TD, PD, DSD, and TRD [5, 6]. Therefore, an important duplication mode (TRD) was missing in the above studies. TRD often leads to the formation of pseudogenes, while other types of duplication lead to rapid expansion of the plant genome [7], resulting in severe functional redundancy and increased functional differentiation within plant gene families [7]. TRD can occur through DNA-based or RNA-based mechanisms. For example, in graminaceous plants, DNA transposons such as packmules (rice (*Oryza sativa* L.)) [8], helitrons (maize (*Zea mays* L.)) [9] and CACTA elements (sorghum (*Sorghum bicolor* L.)) [10] can relocate duplicated genes or gene fragments to new chromosomal locations. Although there are many methods and tools available to perform gene duplication origin analysis [4, 11–13], they do not identify all types of duplication origin due to algorithmic limitations. This may be the reason for the phenomenon of incomplete conclusions. Qiao et al. developed a pipeline named DupGen_finder that can identify all duplication types by optimizing the MCScanX algorithm [5]. This makes it possible to study the complete duplication origin of genes.

The *TLP* family gene was first discovered in the mouse genome [14] and widely exists in animal and plant genomes [15]. TLP protein has a tubby domain (PF01167) of approximately 270 aa at the *C*-terminus, with other possible distinct domains at the *N*-terminus [16–18]. In 1999, Boggon et al. published the crystal structure of the tubby domain, which consists of a hydrophobic α-helix surrounded by a β-barrel structure containing 12 sheets of inverted β-folds. The hydrophobic alpha helix is positioned at the *C*-terminus of the TLP protein [19]. In contrast to the diversity of *N*-terminal structures in animals, the *N*-terminal end of TLP proteins in plants often contains a conserved F-box domain [20].

TLP proteins are essential for growth and development in plants and animals, and deletion of TLP family proteins often leads to changes in the phenotypic characteristics of animals and even to serious diseases. For example, mice deficient in the tubby gene show symptoms such as obesity, blindness, and deafness [14, 21–23]. Mutations in the *TULP1* gene in humans cause an autosomal recessive form of retinitis pigmentosa [24–26]. In a TLP study in *Arabidopsis* (*Arabidopsis thaliana* (L.) Heynh.), Lai et al. found that overexpression of the *AtTLP9* gene in transgenic plants enhanced their sensitivity to ABA [16]. In rice TLP studies, the OsTLP2 protein was found to bind to the PRE4 *cis*-element of the promoter region of the *OsWRKY13* transcription factor, which is induced by pathogens and regulates resistance to bacteria and fungi in rice [27]. Furthermore, many studies on plant *TLP* genes have shown that these genes are involved in plant responses to biotic and abiotic stresses, enhancing plant resistance to a variety of stresses [17, 28, 29].

Previous research has shown that TLPs evolved from an ancestor of the scramblase-like protein family [30]. The sequences corresponding to the tubby domains are conserved in both unicellular and multicellular organisms, but the *N* termini of TLPs are distinct [31]. Intriguingly, plant TLPs have conserved F-box domains in the *N*-terminal sequences, whereas they are highly divergent in animals [27, 28]. In plants, there are more members of the TLP family than in animals [20]. For example, five TLP family members have been identified in vertebrates [22], while 11 family members have been identified in the genome of *Arabidopsis* [16]. Moreover, it has been observed that up to 80% of genes in *Arabidopsis* are the result of lineage-specific expansion [32]. The sequence and architectural similarity of the *TLP* gene family in rice suggests that the rice TLP family may have originated from the same ancestral gene [27]. In cotton, 28 of the 29 paralogous gene pairs have undergone purifying selection [33]. In a comprehensive study of the *TLP* gene family in *Arabidopsis*, rice, and poplar (*Populus trichocarpa* (Torr. and Gray)), *Ka*/*Ks* ratios indicated that most of the duplicated gene pairs experienced positive selection, while some of the remaining gene pairs experienced neutral selection [34]. By using the *Arabidopsis TLP* gene family as a reference, it was found in Brassica that *TLP* genes in *Brassica napus* are not directly amplified compared to those in the diploid parents. Instead, indirect amplification of the *TLP* gene family occurs in the two diploid parents [35].

In research on the Brassica *TLP* gene family, Wang et al. identified five origins of *TLP* duplication using the MCScanX program and found that *TLP* genes in Brassica originated only from WGD and DSD [35]. Based on the above analysis, an important duplication mode (TRD) was missing in this study. Therefore, we hypothesize that the origin of duplication of *TLP* genes may include TRD. Because the *TLP* gene family in the grass family, belonging to the monocots, has not been studied, we selected seven representative grass species for duplication type detection of the *TLP* gene family. Moreover, since the evolutionary trajectory of the grass *TLP* gene family is not yet known, we aimed to fill this gap and provide unique insights into the evolution of the Gramineae *TLP* gene family. We also performed evolutionary analysis of *TLP* gene families in the seven examined grass species using our previously established gene family analysis pipeline [36, 37]. The purpose of this study was to determine whether TRD facilitated the evolution of the *TLP* gene family and to explore the evolutionary footprint of the *TLP* gene family in grass species.

## Results

### The duplication origin modes of *TLP* genes are WGD, DSD, and TRD

**Fig. 1:**
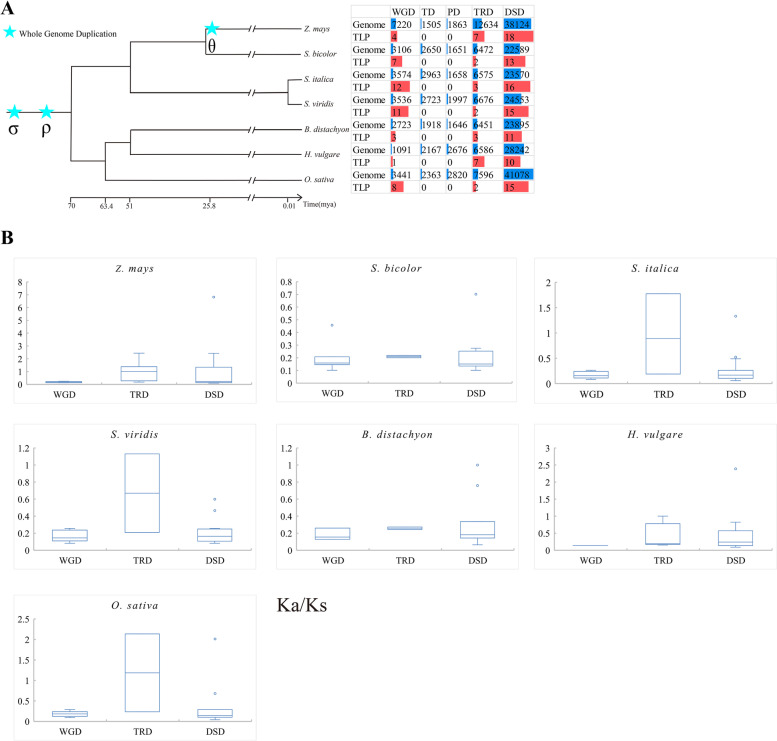
The number and *Ka*/*Ks* ratio distributions of gene pairs derived from different modes of duplication in seven grass species. **A** The number of gene pairs derived from different modes of duplication in seven grass species. Whole-genome duplication (WGD), tandem duplication (TD), proximal duplication (PD), transposed duplication (TRD), and dispersed duplication (DSD). The WGDs that occurred on different branches are labelled. The tree of life and polyploidy information of grass species came from previous reports. **B** The nonsynonymous substitution rate (*ka*)/synonymous substitution rate (*ks*) (*Ka*/*Ks*) ratio distributions of gene pairs derived from different modes of duplication in seven grass species. WGD: whole-genome duplication, TRD: transposed duplication, DSD: dispersed duplication

To investigate whether the duplication origin types of *TLP* genes include TRD, we used the DupGen_finder pipeline to identify all possible duplication types: WGD, TD, PD, DSD, and TRD. WGD and DSD were detected, and no TD and PD were detected (Fig. 1A, Additional file 1: Table S1), which is consistent with the findings of Wang et al.. However, our results showed that the evolution of *TLP* genes was also affected by TRD, as we hypothesized (Fig. 1A, Additional file 1: Table S1). Furthermore, we found that the number of gene pairs with DSD was the highest for the *TLP* gene family in all grass species studied. The number of TRD pairs was greater than the number of WGD pairs in maize and barley (*Hordeum vulgare* L.), equal to the number of WGD pairs in *Brachypodium* (*Brachypodium distachyon* L.), and less than the number of WGD pairs in the other species (sorghum, foxtail millet (*Setaria italica* L.), green foxtail (*Setaria viridis* L.), and rice) (Fig. 1, Additional file 1: Table S1).

**Fig. 2:**
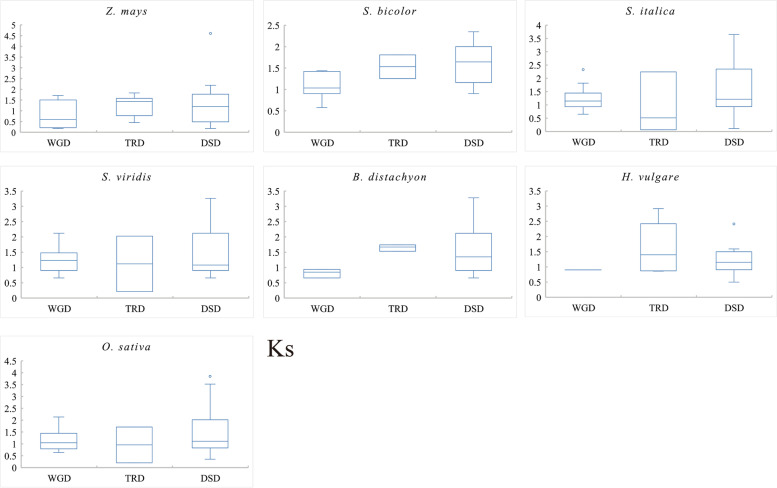
*Ks* distributions of gene pairs derived from different modes of duplication in seven grass species. Whole-genome duplication (WGD), transposed duplication (TRD), dispersed duplication (DSD)

Further calculations of *ka*/*ks* for the three duplicated gene pairs showed that the three duplicates of the seven species generally underwent purifying selection (*ka*/*ks* < 1) (Fig. 1B, Additional file 1: Table S1). In particular, some TRD gene pairs in foxtail millet, green foxtail, and rice were subject to positive selection (*ka*/*ks* > 1). This may be related to the active nature of transposons. The distribution of *Ks* values for the three duplicated gene pairs showed no obvious regularity in the timing of these three duplications (Fig. 2, Additional file 1: Table S1). In general, WGD occurred later than TRD and DSD, with DSD occurring somewhat earlier. Of course, the small amount of current data limits the generality of this conclusion.

### Phylogenetic analysis suggests the phenomenon of duplication and loss

**Fig. 3:**
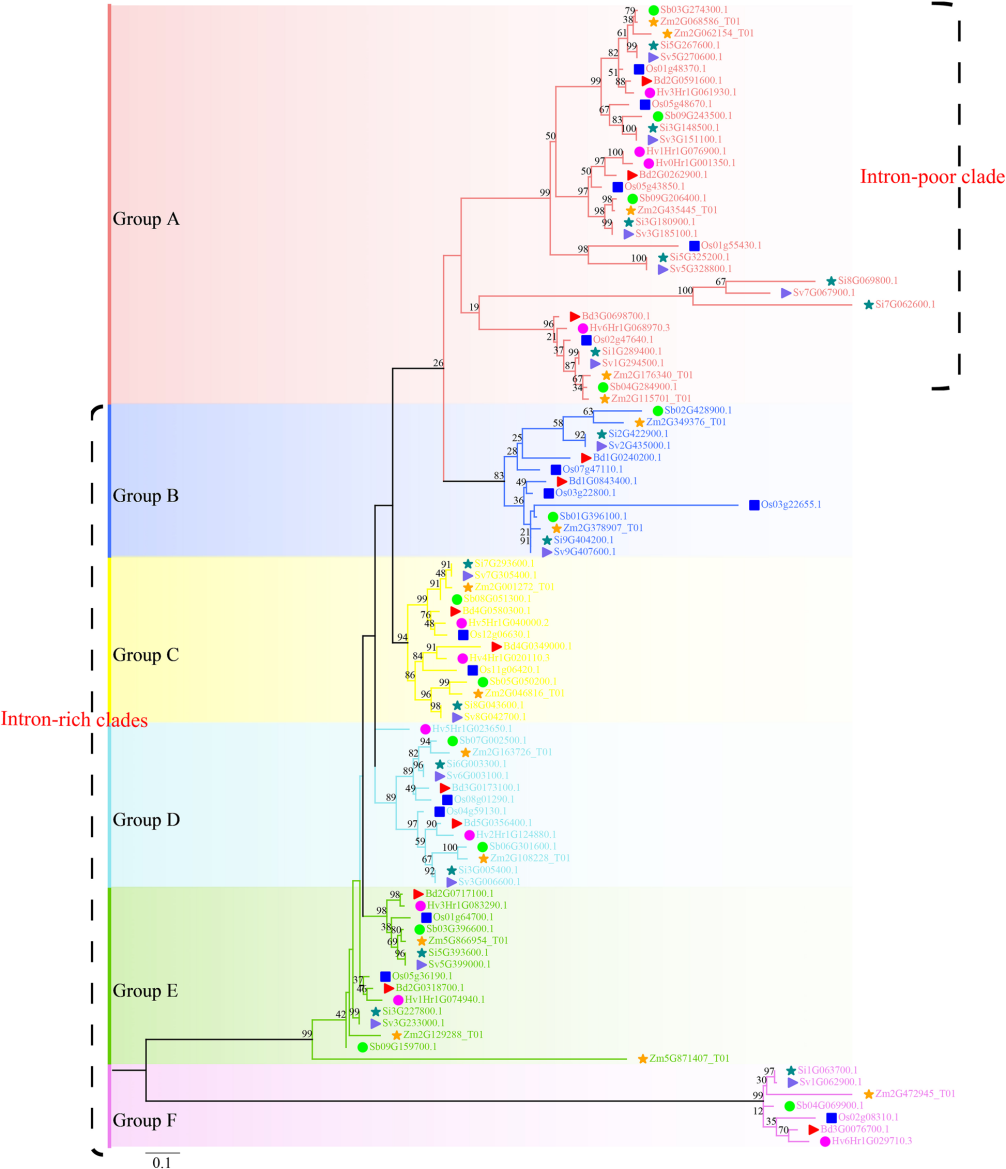
Phylogenetic tree and classification of the TLPs of seven grass species. Here, gene IDs show their respective origin: Zm for maize, Sb for sorghum, Si for foxtail millet, Sv for green foxtail, Bd for *Brachypodium*, Hv for barley, and Os for rice. We used shapes and colours to distinguish different species, with orange stars, green circles, dark green stars, mauve triangles, red triangles, purple circles, and blue squares representing the tubby-like protein (*TLP*) genes in maize, sorghum, foxtail millet, green foxtail, *Brachypodium*, barley, and rice, respectively

A total of 97 *TLP* genes were identified, including 15 in maize, 13 in sorghum, 16 in foxtail millet, 15 in green foxtail, 12 in *Brachypodium*, 11 in barley, and 15 in rice (Fig. 3, Additional file 2: Table S2). Based on Wang et al.’s research and the topology of the tree. The phylogenetic tree of grass TLPs could be divided into six main groups, Group A, Group B, Group C, Group D, Group E, and Group F. Furthermore, Group A was classified as an intron-poor clade, and Groups B-F were classified as intron-rich clades (Additional file 3: Fig. S1, Additional file 4: Table S3).

We observed significant differences between these two clades, as the *TLP* genes in the intron-rich clade maintained a quantitative distribution consistent with the species tree of life, mostly accompanied by the loss of a duplicated maize gene after polyploidy. For example, a copy of *Zm2G472945_T01* was deleted in Group F. However, a copy of *Zm2G129288_T01*, *Zm5G871407_T01*, was retained in Group E. In addition, *Os03g22800.1* showed a duplicate gene, *Os03g22655.1*, but a barley *TLP* orthologue was lost in Group B. Group A in the intron-poor clade was the youngest branch with more divisions. The above analyses suggest that the *TLP* gene family has undergone different degrees of duplication and loss in different grass species.

### Convergence in family size by shrinkage

**Fig. 4:**
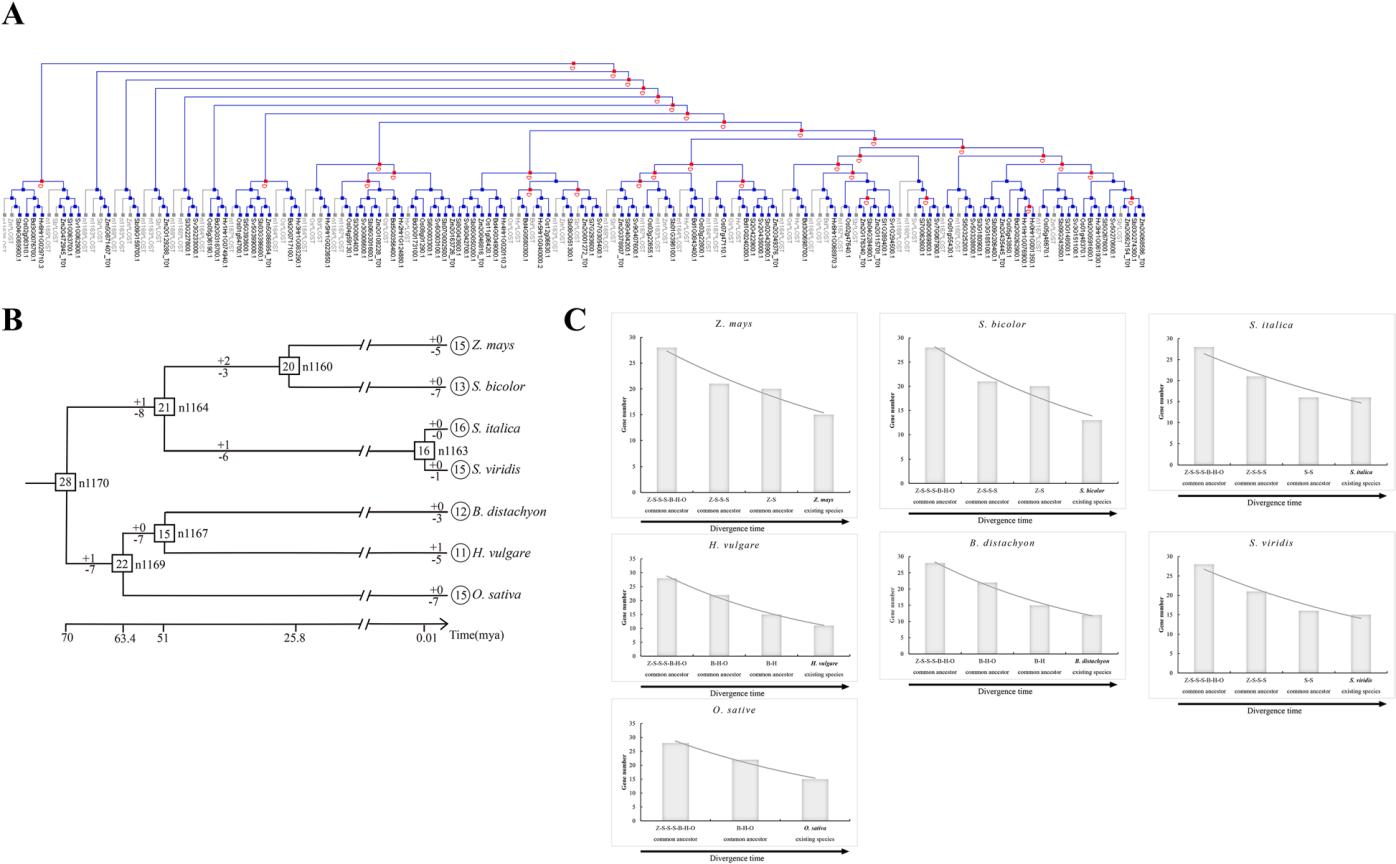
Gain and loss of *TLP* genes in seven grass species. **A** The reconciliation between the species tree and gene tree along with the confirmation of the gene loss/duplication scenario was performed using Notung. The species tree is shown in Fig. 2B. The gene tree is shown in Fig. 1. Red “D”s at branching points indicate predicted gene duplications. Grey branches indicate gene losses. **B** Schematic diagram of gain and loss of tubby-like protein (*TLP*) genes in seven grass species. The numbers in the rectangles and circles represent the number of *TLP* genes in ancestors and existing species. The + and − signs represent the gain and loss of genes, respectively. **C** Evolutionary patterns of *TLP* genes in seven grass species. Z-S-S-S-B-H-O indicates the common ancestor of all seven grass species; Z-S-–S-S indicates the common ancestor of maize, sorghum, foxtail millet, and green foxtail; Z-S indicates the common ancestor of maize and sorghum; S-S indicates the common ancestor of foxtail millet and green foxtail; B-H-O indicates the common ancestor of *Brachypodium*, barley, and rice; and B-H indicates the common ancestor of *Brachypodium* and barley

Based on the phylogenetic tree, it could be inferred that many independent gene gains and losses occurred during different stages of grass evolution (Fig. 4A). We found that the *TLP* gene family shrank after the WGD event common to grasses ~ 100 million years ago (Fig. 4B). The analysis of gene increases and decreases based on the gene phylogeny showed that after the grass-wide WGD event, there should have been at least 28 *TLP* genes in the grass common ancestor (Fig. 4B). Starting with 28 ancestral genes, the family size of each sublineage then gradually decreased over time to result in the family size of the extant species. For instance, the common ancestor of *Brachypodium*, barley, and rice had 22 *TLP* genes after having gained one gene and lost seven genes. After that, rice had 15 *TLP* genes, gaining zero genes and losing seven genes (Fig. 4B, C). Similar decreases in family size were found in the other studied grasses (Fig. 4B, C). Thus, the grass species that we studied exhibit a “consistent shrinking” evolutionary pattern.

### Synteny network and phylogenomic analyses of TLPs reveal duplication events

**Fig. 5:**
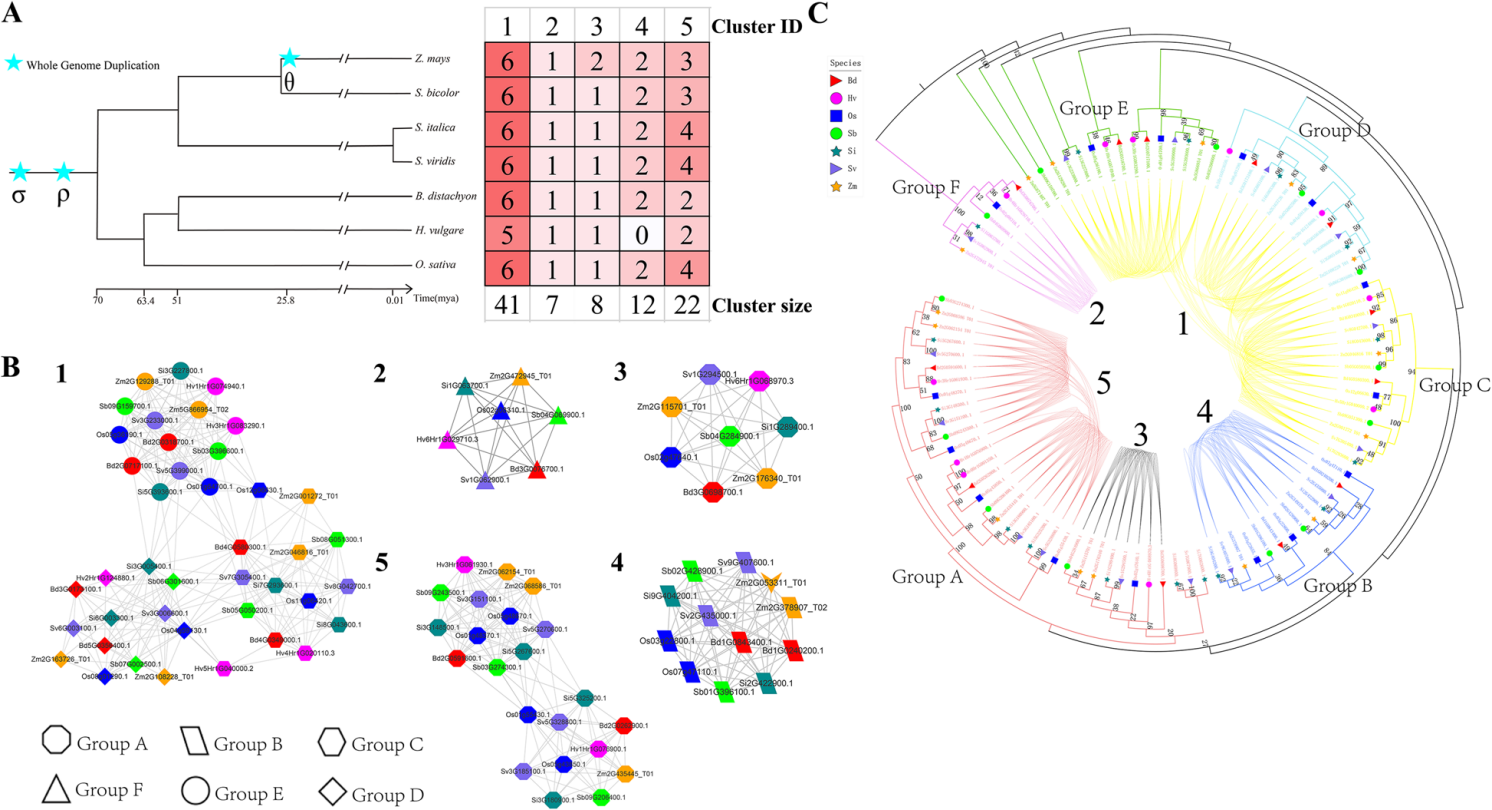
Synteny network clusters and phylogenetic profiling of the *TLP* gene families in seven grass species. **A** Species composition for each of the five network communities. Red-coloured cells depict the presence of tubby-like protein (*TLP*) syntelogs (syntenic homologous genes) in the different species. The five network communities were identified using CFinder at k = 3. The cluster ID and size are indicated at the top and bottom, respectively. The tree of life and polyploidy information of grass species came from previous reports. **B** Detailed visualization for each of the TLP synteny network communities. Nodes in different colours represent different grass species, and the different node shapes represent the different groupings (Groups A to F) belonging to the phylogenetic tree. The V shape indicates that the gene does not belong to the *TLP* gene family. **C** Maximum-likelihood gene tree for the *TLP* gene family and syntenic relationships between the genes. Each connecting line located inside the inverted circular gene tree (implemented in iTOL) indicates a syntenic relationship between two *TLP* genes (syntelogs). This phylogenetic tree is consistent with Fig. 1, and the numbers 1–5 are cluster IDs, which are consistent with **A**,** B**

Phylogenomic synteny network analyses can identify the different types of duplicates from WGDs or TDs on the basis of phylogenies [38, 39]. To further understand the duplication history of TLPs, we performed synteny network and phylogenomic analyses of TLPs. We found that the synteny network of grass TLPs contained five clusters, named Cluster 1, Cluster 2, Cluster 3, Cluster 4, and Cluster 5, with sizes of 41, 7, 8, 12, and 22, respectively (Fig. 5A, B, Additional file 5: Table S4). Duplications and losses of several genes were found; for example, one maize *TLP* gene from Cluster 3 was duplicated during the theta (θ) WGD event, and two barley *TLP* genes from Cluster 4 were lost.

According to the phylogenetic tree of the *TLP* genes, the genes of Clusters 1, 2, and 4 belong to the intron-rich clade, and the genes of Clusters 3 and 5 belong to the intron-poor clade (Figs. 3 and 5B, C). We found a high degree of consistency between the gene sets in each cluster and the phylogenetic tree. For example, Cluster 2 genes were all contained in Group F, and Cluster 1 genes were contained in adjacent Groups E, D, and C (Fig. 5B, C). The above phenomenon implies that the evolutionary pattern of grass *TLP* genes is one of conservation.

### Homology clustering reflects species proximity and strong purifying selection

**Fig. 6:**
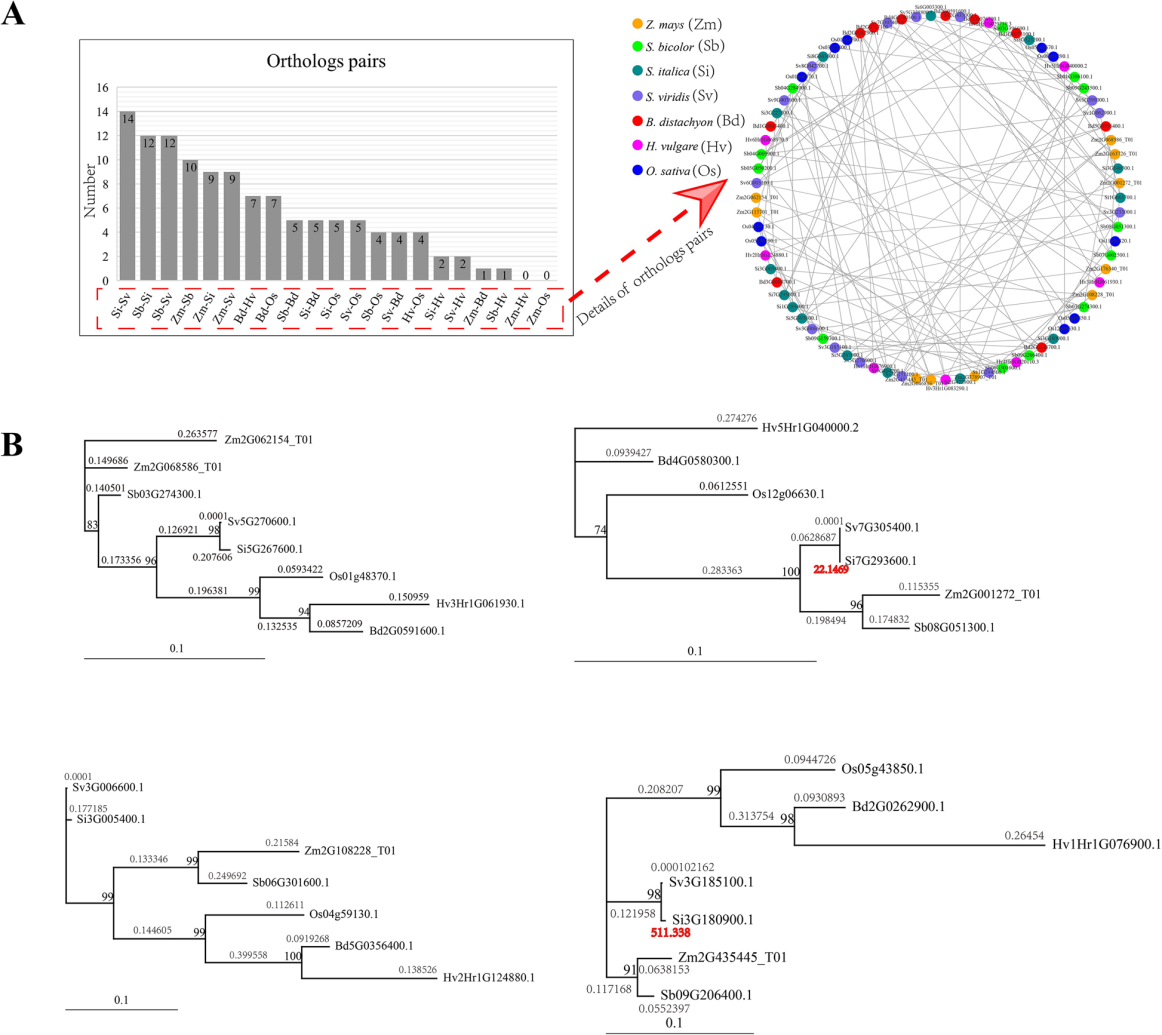
Orthologous network and selection pressure analysis of *TLP* genes in seven grass species. **A** Quantitative distribution of tubby-like protein (*TLP*) orthologous gene pairs in seven grass species. Nodes in different colours represent different grass species. **B** Maximum likelihood gene tree and selection pressure of single-copy genes clustered by the Markov cluster (MCL) algorithm. A nonsynonymous substitution rate (*ka*)/synonymous substitution rate (*ks*) (*ka*/*ks*) less than 1 indicates purifying selection, and a *ka*/*ks* greater than 1 indicates positive selection. The range for all values of *ka*/*ks* less than 1 is 0.0001 to 0.399558, and values of *ka*/*ks* greater than 1 are highlighted in red

The identification of homologous genes in grasses showed that foxtail millet had the most orthologous gene pairs with the *TLP* gene family of green foxtail, and maize had the fewest orthologous gene pairs with the *TLP* gene families of barley and rice (Fig. 6A, Additional file 6: Table S5). This reflects the fact that the more closely related to each other the species are, the more *TLP* homologous gene pairs there are. The MCL homology clustering of grass *TLP* genes yielded 16 classes. Among them, there were four clusters containing genes from each species, and they showed single-copy gene patterns (Fig. 6A, Additional file 7: Table S6). Phylogenetic tree reconstruction and selection pressure on these four single-copy gene clusters showed that a total of 41 branches (95.35%, 43 branches in total) were subject to strong purifying selection (the ratio of the nonsynonymous to synonymous distances (ω) ranged from 0.0001 to 0.399558) (Fig. 6B), suggesting that grass *TLP* gene evolution has involved strong purifying selection.

### Codon bias is weak and similar

**Fig. 7:**
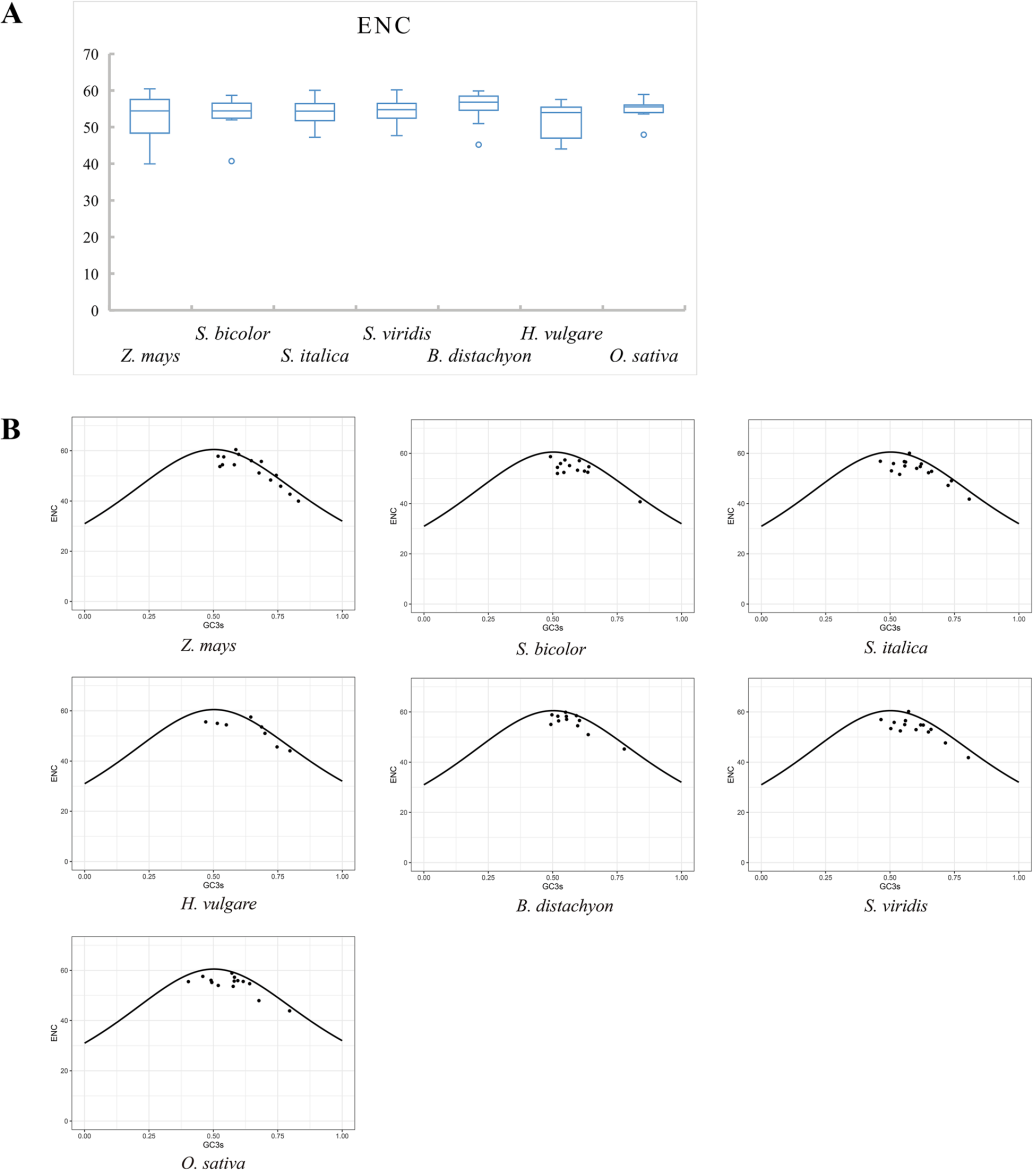
Codon bias and ENC-GC3s plots of seven grass *TLP* gene families. **A** Box plot of ENC values for seven grass tubby-like protein (*TLP*) gene families. ENC indicates the number of valid codons. The value of ENC ranges from 20 to 61. An ENC value less than 35 indicates strong codon bias. **B** ENC-GC3s plots of seven grass *TLP* gene families. The ENC-GC3s plot was used to evaluate the influence of mutation selection on codon bias. GC3s indicates the GC content at the third codon site. The horizontal axis represents the GC3s value, and the vertical axis represents the ENC value

To investigate whether codon usage bias has contributed to *TLP* gene evolution, we performed codon bias analysis on the *TLP* genes of each species using the CodonW program. The value of ENC denotes the number of effective codons and varies from 20 to 61; an ENC value less than 35 indicates strong codon bias [40, 41]. Our results show that the ENC values for the *TLP* gene family in each species are greater than 39 (Fig. 7A, Additional file 8: Tables S7, Additional file 9: S8), suggesting that the codon bias of the *TLP* genes is very weak.

ENC-GC3s plots were used to evaluate the influence of mutation selection on codon bias [40, 42]. Most of the *TLP* genes of the seven species were below and away from the standard curve (Fig. 7B), indicating a large difference between their actual and expected ENC values, suggesting that base mutations are not the main factor influencing their codon bias but that they may also be influenced by natural selection and other factors. A small number of genes were located at and near the standard curve, indicating that their actual ENC values were close to the expected values and suggesting that their codon bias is influenced by base mutations. Therefore, in conjunction with the previous selection pressure analysis, these findings indicate that base mutation and selection pressure jointly promoted the codon bias of *TLP* genes in the seven grass species, and purifying selection pressure may have a greater impact.

**Fig. 8:**
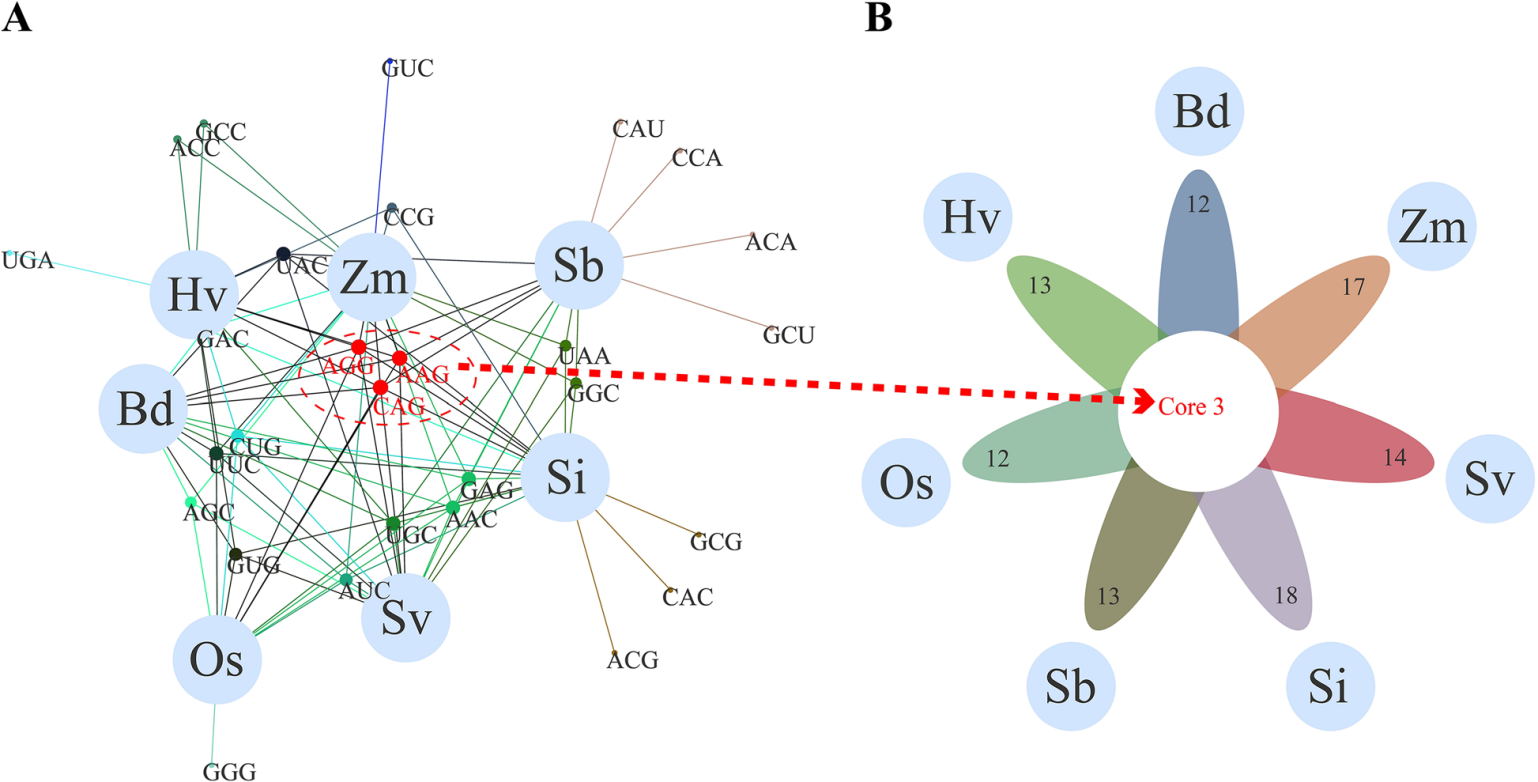
Optimal codon analysis of seven grass *TLP* gene families. **A** Venn network map of the optimal codons of the seven grass tubby-like protein (*TLP*) gene families drawn by Evenn. The differently coloured lines represent the optimal codons shared by different species combinations. Optimal codons shared by the seven grass *TLP* gene families are highlighted in red. Zm for maize, Sb for sorghum, Si for foxtail millet, Sv for green foxtail, Bd for *Brachypodium*, Hv for barley, and Os for rice. **B** A flower plot of the optimal codons of the seven grass *TLP* gene families. Zm for maize, Sb for sorghum, Si for foxtail millet, Sv for green foxtail, Bd for *Brachypodium*, Hv for barley, and Os for rice

Regarding optimal codons, maize has 17 optimal codons, 1 of which ends in A and 16 of which end in G/C; sorghum has 13 optimal codons, 5 of which end in A/U and 8 of which end in G/C; foxtail millet has 18 optimal codons, 1 of which ends in A and 8 of which end in G/C; green foxtail has 14 optimal codons, 1 of which ends in A and 13 of which end in G/C; *Brachypodium* has 12 optimal codons, 0 of which end in A/U and 12 of which end in G/C; barley has 13 optimal codons, 1 of which ends in A and 12 of which end in G/C; and rice has 12 optimal codons, 0 of which end in A/U and 12 of which end in G/C (Fig. 8, Additional file 10: Table S9). These results imply that the optimal codon of the *TLP* gene family in all seven species prefers to end in C or G.

Venn network analysis showed that the seven grass *TLP* gene families share three optimal codons (AGG, AAG, and CAG). Maize shares a single optimal codon (GUC), sorghum shares four optimal codons (CAU, CCA, ACA, and GCU), grain shares three optimal codons (GCG, CAC, and ACG), barley shares one optimal codon (UGA) and rice shares one optimal codon (GGG) (Fig. 8, Additional file 11: Table S10). In summary, all results indicate that the codon biases of the seven species are nearly identical.

## Discussion

### Five mechanisms for the origin of gene duplication

MCScanX is often used to identify the type of origin of gene duplication in the evolutionary analysis of gene families [1–3, 35, 41, 43–53]. However, the duplication types identified by the duplicate_gene_classifier program of MCScanX lack the TRD type, and it is enforced that a gene can originate from only one duplication type. Therefore, caveats and limitations need to be recognized, presented, and discussed when interpreting the results from MCScanX.

Genes can be duplicated through a variety of mechanisms, except for WGD, which are collectively referred to as small-scale duplication/single gene duplication. TD copies are consecutive in the genome, and copies from PD are close to each other but separated by several genes. These two patterns of gene duplication are thought to arise through unequal crossing over or localized transposon activities. DSD copies are not contiguous within genomes and homologous chromosome segments. Distant single-gene transposition can explain the widespread dispersed duplication within and among genomes. TRD may occur through DNA-based or RNA-based mechanisms. DNA-based mechanisms occur by relocating the copied gene or gene fragment to a new chromosomal locus via DNA transposons. RNA-based transposed duplication works by reverse transcription of spliced messenger RNA to produce a single-exon retrocopy from a multi-exon parental gene. The new retrogene is deposited in a new chromosomal environment with new (i.e., nonancestral) neighbouring genes [5, 6, 54]. Therefore, the identification of transposed duplication requires the assistance of a reference genome. Based on this principle, Qiao et al. developed an analytical pipeline (DupGen_finder) to address the above issues by optimizing the MCScanX algorithm, opening up avenues for researchers to comprehensively analyse patterns of gene duplication origins.

### The evolution of *TLP* genes was influenced not only by whole-genome duplication and dispersed duplication but also by transposed duplication

Previous studies have shown that *TLP* family genes in Brassica originated only from WGD and DSD [35]. Here, we perform a series of evolutionary analyses on the *TLP* gene family in grasses. The *TLP* genes were found to have originated not only from WGD and DSD but also from TRD, which has been neglected. Previous studies on model plants have shown that WGD and TD contribute most to genetic redundancy, while other duplication modes contribute more to evolutionary novelty. Among them, inferred transposon-mediated gene duplication tends to reduce gene expression levels [6]. We found that *TLP* genes are subject to TRD, and therefore, this may affect *TLP* gene expression. However, this requires complex experiments for verification.

A plant gene family is a group of genes with related functions that arise from a single copy of an ancestral gene source through gene duplication and retain similar sequences and structures. Based on the extent of duplication, the size of the duplication region and the impact of transposons, gene families can be associated with duplication events such as TD, WGD, or TRD. The direct result of gene duplication may be functional differentiation of genes, including subfunctionalization, neofunctionalization, pseudogenization, and concerted evolution [55]. Evolutionary studies of genes can provide important clues to explain the functional differentiation of genes. Consequently, for the evolutionary analysis of a gene family, it is essential to parse the history of duplication experienced by the family. This is the main reason why in recent years, whenever the evolution of a gene family was considered, the type of origin of gene duplication was analysed. Herein, our study successfully demonstrates a complete gene family duplication origin analysis, and our results provide new insights into the evolution of the *TLP* gene family.

### The “consistent shrinking” evolutionary pattern for the *TLP* gene family in grasses

A recent large-scale survey of plant gene family evolution showed that gene duplication and gene loss occurred in almost all gene families during plant evolution [7]. The same family evolutionary history was found in our study. Furthermore, we found that the grass *TLP* gene family underwent more loss than duplication, achieving similar gene numbers through constant shrinkage (Fig. 4). This evolutionary pattern of the *TLP* gene family may be beneficial to grasses, but the exact benefits remain to be explored.

Genes generate functional innovation through multiple duplication mechanisms and contribute to the adaptive evolution of species [55–58]. Nevertheless, plants often do not need very many copies of genes, as more copies may complicate the regulatory pathways and more homologues, mutated or intact, can induce conflicts and lead to plant death [37]. Many studies have shown that there is significant loss of new genes as a result of duplication and that the rate of gene loss tends to be inconsistent from species to species [59–61]. In our study, the synteny network (Fig. 5) and the homology network (Fig. 6) showed the loss of gene copies caused by WGD.

### Base mutation and selection pressure jointly promoted the codon bias of *TLP* gene families

The drivers of genetic evolution, such as natural selection and base mutations, are diverse. Our study shows that the evolution of *TLP* genes mainly involved purifying selection (Figs. 1B and 6B), which is consistent with the evolutionary pattern of most genes [36, 41–43, 56, 62]. Codon usage bias has been hypothesized by some to have contributed to adaptive gene evolution [63]. Our codon bias analysis of *TLP* showed that base mutation and selection pressure jointly promoted the codon bias of grass *TLP* genes, and purifying selection pressure may have had a greater impact. Moreover, we found that the codon biases of the *TLP* gene family were consistent across the seven grass species. Optimal codons usually have high expression levels and can therefore be used as one of the codon bias parameters, providing a basis for codon modification during later transgenesis [41]. Our study showed that the optimal codon numbers of the seven grass *TLP* gene families ranged from 12 to 18, and these small numbers reflected the effects of purifying selection and mutational pressure [64]. Moreover, previous research findings have shown that GC content elevation results in codon usage bias [56, 65]. The majority of the optimal codons of the grass *TLP* family contain GC bases, which may be evidence that codon usage bias is related to GC content.

## Conclusions

In summary, by selecting the grass *TLP* gene family for duplication origin type analysis, we supported our conjecture that *TLP* genes originated from TRD in addition to the previously reported WGD and DSD. Moreover, our research shows that the evolution of the *TLP* gene family is at least affected by forces such as duplication, natural selection, and base mutations. We hope that our work can provide a reference for complete studies of gene duplication patterns and advance our understanding of the evolution of the *TLP* gene family.

## Materials and methods

### Collection of data and identification of *TLP* genes

Based on previous studies [37, 58], rice, maize, sorghum, foxtail millet, green foxtail, *Brachypodium*, and barley can be considered representative species for Poaceae lineages. Therefore, we obtained genomic and annotation data for these seven Gramineae species from public databases. First, the latest proteome of rice (version 7.0) was obtained from the dedicated rice database (http://rice.uga.edu/) [66]. The corresponding data for the remaining representative species of grasses were downloaded from the Phytozome database (https://phytozome-next.jgi.doe.gov/) [67] [*Zea mays Ensembl-18*, *Sorghum bicolor Rio v2.1*, *Setaria italica v2.2*, *Setaria viridis v2.1*, *Brachypodium distachyon Bd21-3 v1.1*, and *Hordeum vulgare r1*]. A hidden Markov model for the tubby domain of TLP (PF01167) was downloaded from the Pfam database [18]. HMMER software [68] was then used to identify proteins in the proteome that contained tubby domains (*e* values less than 1*e*-10), and these candidates were further identified by the Pfam, NCBI-CDD [69], and SMART [70] databases. To identify family members as comprehensively as possible, we employed the blastp program (*e* values less than 1*e*-10) to search for all possible TLP family members in the proteomes of these seven grass species, using the amino acid sequence of rice TLPs as a reference. All candidate family members were then confirmed through the domain database above. Finally, the members identified by blastp were identical to those identified by HMMER. In addition, files required for subsequent analyses were downloaded from the corresponding databases, including General Feature Format Version 3 (GFF3) and coding sequence (CDS) files.

### Identification of *TLP* gene duplications

The different modes of gene duplication were identified using the DupGen_finder pipeline developed by Qiao et al. [5]. The DupGen_finder pipeline was used for the specific identification of gene pairs corresponding to the five duplication types in a species (see Additional file 12: Fig. S2). First, gene pairs with the five duplication types were identified within the whole genomes of all species, including WGD, TD, PD, TRD, and DSD, using *Spirodela polyrhiza* as the outgroup for monocot plants (the latest proteome and GFF3 annotation files for *S. polyrhiza* were obtained from Phytozome), according to previously described methods and criteria [4, 5, 71, 72]. Then, a custom Python script (Additional file 13: Program S1) was used to extract all duplication pairs of *TLP* for each species.

### Calculation of nonsynonymous (*ka*) and synonymous substitutions (*ks*)

A previously published custom Perl program was used to calculate *ka* and *ks* for duplicate gene pairs using the BioPerl module, the ClustalW program, the NG method, and a Poaceae evolution rate of 6.5 × 10^–9^ [36, 41, 73, 74]. The boxplots of *Ka*/*Ks* and *Ks* for different types of duplicated gene pairs in the seven grass species were drawn by MS Excel.

### Construction of a phylogenetic tree

First, full-length amino acid sequences of the TLPs of all grasses were aligned in MUSCLE using default parameters [75]. Then, the Jones–Taylor–Thornton + gamma distributed (JTT + G) model was determined to be the best model via MEGA X [76]. Finally, MEGA X was employed to construct maximum likelihood (ML) trees with the above model and 1000 bootstrap replicates.

According to the number of introns, eukaryotic genes can be divided into three categories: intronless (no introns), intron-poor (three or fewer introns per gene), and intron-rich (more than three introns per gene) [2]. Thus, the number of introns for each *TLP* gene was calculated by CFVisual software [77] based on GFF3 files and characterised into different categories. Finally, based on the distribution of these categories of genes in the phylogenetic tree, Adobe Illustrator (Ai) was used to delabel the intron-poor clade and intron-rich clade of the phylogenetic tree. In addition, the tree of life and polyploidy information of grass species in this study came from previous reports [36, 58, 78].

The constructed phylogenetic tree of the *TLP* gene family was reconciled with the species tree of life using Notung software [79] to infer duplication and loss events of *TLP* genes in grasses. Finally, the number of *TLP* family genes for each ancestral node was back-projected from the family gene size of the extant species [36, 80–82].

### Construction and clustering of the synteny network and phylogenetic profiling of clustered communities

We used the synteny network (Synet) method developed by Zhao et al. for syntenic block calculations, network construction, and community detection [38]. First, pairwise all-against-all comparisons were performed using Diamond [83] with default settings [84] for whole-genome proteins of each of the seven grasses. Then, MCScanX was used to compute genomic collinearity between all pairwise genome combinations using default parameters (minimum match size for a collinear block = 5 genes, maximum gaps allowed = 25 genes) [38, 85]. Then, the output files from all the intra- and interspecies comparisons were integrated into a single file named “SynNet-k5s5m25”. Finally, a custom Python script (Additional file 14: Program S2) was used to extract the Synet of the grass *TLP* gene family according to Zhao et al.’s criteria; rows containing at least one family gene were retrieved into subnetworks [38]. Clique percolation as implemented in CFinder [86–88] was used to locate all possible k-clique communities for the TLP synteny network to identify communities (clusters of gene nodes) [38]. Then, the clustering results of the TLP synteny network (k = 3) were visualized in Cytoscape [89] to depict an undirected and unweighted network [39]. Finally, gene pairs in different clusters of the TLP synteny network were linked in a phylogenetic tree of the grass *TLP* gene family using iTOL [90] to perform phylogenetic profiling of clustered communities with differently coloured Bezier curves.

### Inference of orthologues and selection pressure of single-copy gene clusters

The homology of the grass TLP family was inferred using OrthoMCL software (default parameters) [91]. Then, Cytoscape software was used to draw the lineal orthologous relationship network. Clustering analysis was performed using the Markov cluster (MCL) algorithm (− I > 1.5) [45, 92]. Single-copy clustered genes were used to construct the ML phylogenetic tree via FastTree [93], and the trees were then used to perform further maximum likelihood analysis using the Codeml program in PAML [94]. To detect whether a specific *TLP* gene had been positively selected, we compared two types of competing models, a free ratio model and a ratio-restriction model [95], following our previous steps [36, 56].

### Codon bias and determination of optimal codons

To ensure the accuracy of the analysis, we analysed only the given CDSs with ATG (AUG) as the start codon, for which TAG (UAG), TGA (UGA), or TAA (UAA) was the stop codon, whose length was at least 300 bp and in which only A, T, C, and G bases were present [41]. The CodonW program (https://sourceforge.net/projects/codonw/) was used to analyse codon bias (default parameters), and the ENC-plot was created using the R package to detect the effect of base composition on codon bias, with the standard curve calculated as ENC = 2 + GC3s + 29/[GC3s2 + (1—GC3s)2] [40, 96]. Optimal codons for each species were determined as described in our previous studies [41]. Finally, optimal codons shared by different species were mined by Evenn [97].

## Supplementary Information

**Additional file 1: Table S1.** Patterns of *TLP* gene duplication origins and *Ka*/*Ks* in seven grass species.

**Additional file 2: Table S2.** List of *TLP* genes in seven studied grass species.

**Additional file 3: Figure S1.** Exon-intron distribution of all TLP genes in the seven grasses.

**Additional file 4: Table S3.** Basic statistical details on structural elements of all *TLP* genes in seven grass species.

**Additional file 5: Table S4.** Synteny network of TLPs in seven grass species.

**Additional file 6: Table S5.** Orthologous network of TLPs in seven grass species.

**Additional file 7: Table S6.** The cluster of *TLP* genes in seven grass species obtained using the MCL algorithm.

**Additional file 8: Table S7.** Analysis of codon bias of *TLP* genes in seven grass species.

**Additional file 9: Table S8.** Detailed parameter values for codon bias of eligible *TLP* genes in seven grass species.

**Additional file 10: Table S9.** Optimal codons of *TLP* gene families in seven grass species.

**Additional file 11: Table S10.** Venn calculation results of optimal codons for seven grass species.

**Additional file 12: Figure S2.** The flowchart of DupGen_finder pipeline.

**Additional file 13: Program S1.** Extraction of duplication gene pairs of family genes from all duplication gene pairs of a certain type in a species.

**Additional file 14: Program S2.** Extraction of synteny networks of family genes from genome-wide synteny networks of all studied species.

## Data Availability

The datasets generated and/or analysed during the current study are available in this published article and the additional files. *Oryza sativa* L. genome: the Rice Genome Annotation Project (RGAP) (http://rice.uga.edu/). *Zea mays* Ensembl-18, *Sorghum bicolor* Rio v2.1, *Setaria italica* v2.2, *Setaria viridis* v2.1, *Brachypodium distachyon* Bd21-3 v1.1, and *Hordeum vulgare* r1 genome: the Phytozome database (https://phytozome-next.jgi.doe.gov/).
