# Supplementary figures and images for: Whole-genome and dispersed duplication, including transposed duplication, jointly advance the evolution of TLP genes in seven representative Poaceae lineages
Source: BMC Genomics. 2023 May 30;24:290. doi: 10.1186/s12864-023-09389-z (PMC10228137; doi:10.1186/s12864-023-09389-z)

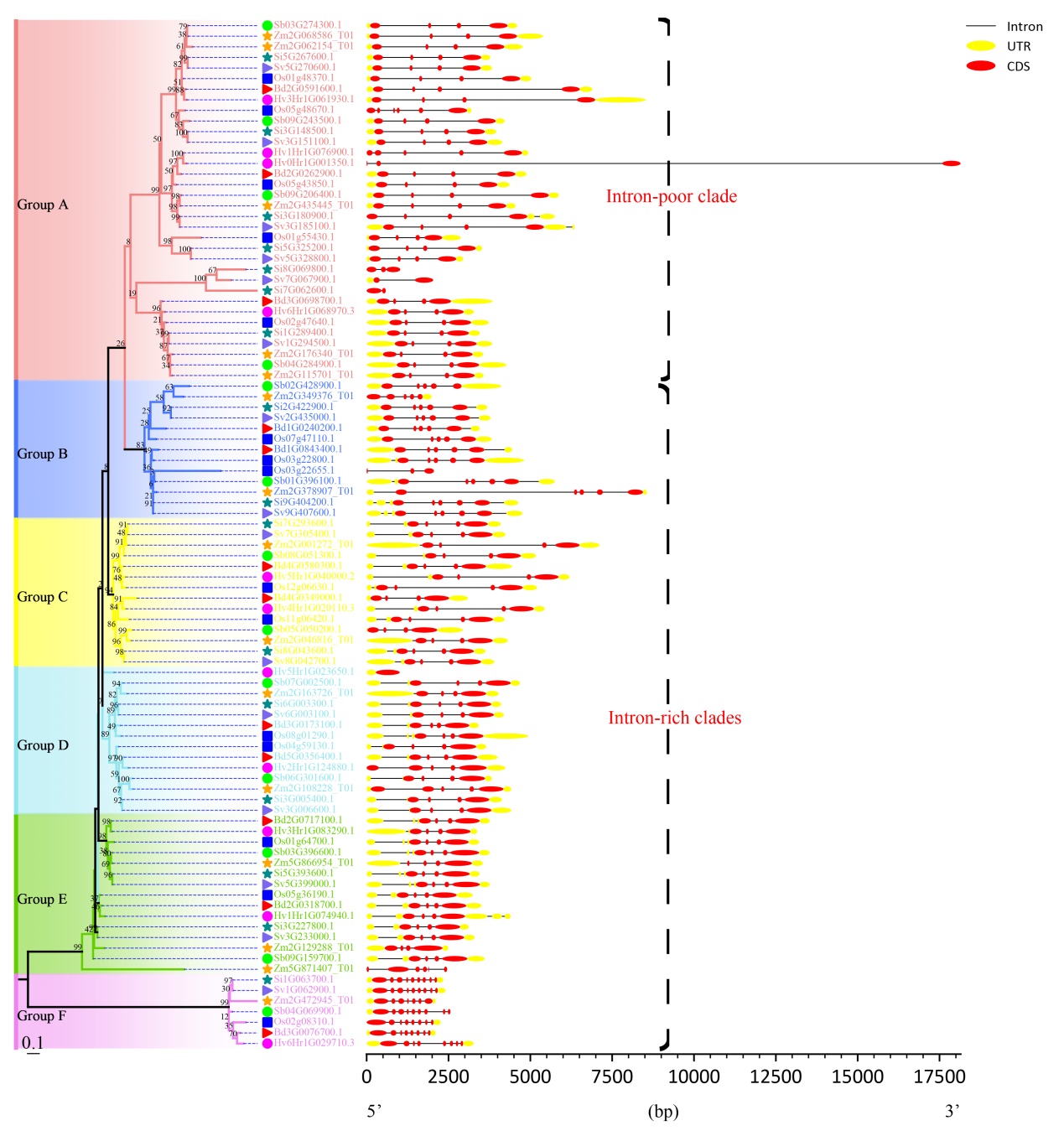


**Figure S1.** Exon-intron distribution of all TLP genes in the seven grasses.

Supplement: Supplementary file 3 — Additional file 3: Figure S1. Exon-intron distribution of all TLP genes in the seven grasses. [file 12864_2023_9389_MOESM3_ESM.docx]

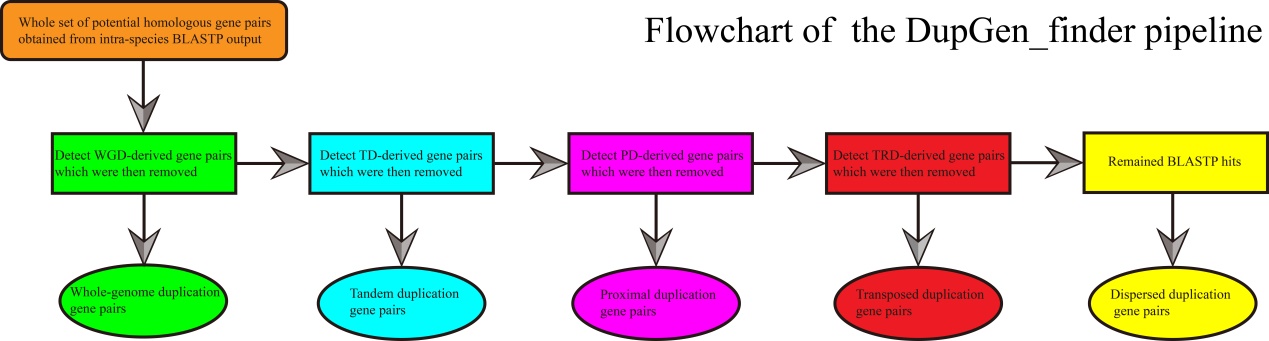


**Figure S2.** The flowchart of DupGen_finder pipeline.

Supplement: Supplementary file 12 — Additional file 12: Figure S2. The flowchart of DupGen_finder pipeline. [file 12864_2023_9389_MOESM12_ESM.docx]
